# Neuralgia over the Spine of Certain Vertebræ

**Published:** 1864-11

**Authors:** 


### NEURALGIA OVER THE SPINES OF CERTAIN VERTEBRÆ.

M. Trousseau observes in *L'Union Medicale*, during a clinic on neuralgia, that when it occupies the branches of the trifacial, it is always at the point of emergence of the ophthalmic branches, of the superior maxillary and of the inferior maxillary that the pain is most acutely felt. Then comes the frontal point where pain rarely fails, then the parietal point where it is frequently wanting, last of all the occipital nerve although not related to the lingual in origin, is almost always affected. He has observed an inexplicable and invariable thing in all cases recorded, that whether the trifacial alone was attacked or the occipital simultaneously affected, pressure on the spinus apophyses of the first two cervical vertebræ was *always* very painful and in a certain number of cases immediately awakened pain in the affected nerves. If the nerves of the brachial plexus were attacked, pressure over the spinous apophyses of the last cervical vertebræ produced pain and it was the same when he explored the vertebral column in the case of intercostal, lumbar and sciatic neuralgia. M. Trousseau lays it down as a rule, that in the various neuralgias the spinous apophyses are painful at a point nearly corresponding to that at which the nerve emerges, and not unfrequently the pain extends a little higher up the vertebral column.

New Anæsthetics.—The *Intellectual Observer* mentions that Dr. Genges has addressed a note to the French Academy detailing some very interesting experiments performed by him in this direction. He has ascertained that a purified kerosoline, obtained from commercial petroleum oil, when vaporized by means of heat, will be found a valuable anæsthetic. He especially recommends as safer than chloroform, brom hydric ether, which not only is less inflammable than ordinary ethers, but possesses an exquisite odor.

*New Use of Electricity.*—The *Medical and Surgical Reporter*, of Philadelphia, describes a new application of electricity to household use, which promises to be of great public utility. The *electric bracket*, by Robert Cornelius, of that city, is designed as an attachment to the ordinary gas burner, "by which the gas may be lighted at any moment by the instantaneous production of a spark of electricity." It consists of an instrument by which simple friction of two surfaces is furnished by a movement as simple and easy as the turning of a key. The apparatus is attached to the bracket, and connected with the gas burner by a fine copper wire covered with silk, and terminating in a platinum point one-sixteenth of an inch from the aperture of the burner. Merely lifting a rubber plug from its bed in a cup lined with lamb's wool a..d silk, which constitutes the apparatus, produces a spark which darts from the platinum point to the burner and ignites the escaping gas.

